# A camera calibration method driven by unmanned aerial vehicles and high-precision estimation of maize SPAD values under adverse conditions

**DOI:** 10.3389/fpls.2026.1922896

**Published:** 2026-09-07

**Authors:** Jianwen Yan, Shilong Miao, Xianyue Li, Haibin Shi, Shijie Ding, Zhen Li, Ning Wang

**Affiliations:** 1 National Key Laboratory of Ecological Environment for Soil and Water Engineering in Arid Areas, Inner Mongolia Agricultural University, Hohhot, China; 2 Inner Mongolia Autonomous Region Engineering Research Center for High-Efficiency Water-Saving Technology Equipment and Water and Soil Environmental Effects, Hohhot, China; 3 Inner Mongolia Autonomous Region Water Resources Research Institute, Hohhot, China

**Keywords:** camera, maize SPAD value, spectral index (SI), texture feature (TI) machine learning, unmanned aerial vehicle (UAV) remote sensing

## Abstract

Rapid and non-destructive monitoring of maize SPAD is important for precision water and nitrogen management in arid irrigation areas. This study developed a UAV–smartphone cross-device calibration framework for SPAD estimation using UAV multispectral imagery, smartphone RGB images, and ground-measured SPAD data collected at the jointing, tasseling, and grain-filling stages. Spectral and texture features were integrated for UAV-based modeling, and regularized least-squares calibration was used to map smartphone-derived features into the UAV feature space. The random forest model using combined spectral and texture features achieved the best UAV-based performance, with validation R² values of 0.81, 0.79, and 0.75 across the three growth stages, representing an improvement of more than 9% over single-feature models. After cross-device calibration, smartphone-based SPAD estimation achieved R² values of 0.80, 0.86, and 0.82, respectively. These results demonstrate that cross-device feature calibration can effectively bridge UAV multispectral and smartphone RGB observations, providing a low-cost and accurate approach for field-scale maize SPAD monitoring and precision water–nitrogen management.

## 1 Introduction

Maize is one of the world’s three major food crops, and its photosynthetic efficiency and nutritional status directly determine its yield. The SPAD value, a core parameter representing the relative chlorophyll content, is a key indicator of the physiological state and stress resistance of maize (Nuss and Tanumihardjo, 2010; Szulc et al., 2021). The Yellow River irrigation district is an important agricultural production base in the arid and semi-arid regions of northwestern China (Li et al., 2017). Given the dual challenges of low annual precipitation (only 168 mm) and poor soil quality, the accurate monitoring of maize SPAD values is of great practical significance for optimizing water and fertilizer allocation and improving resource-use efficiency (Eslamian et al., 2017). Traditional SPAD monitoring relies on handheld devices. Although these methods provide accurate point-based measurements, they are time-consuming and labor-intensive and are inherently limited in spatial coverage, thus failing to achieve large-scale synchronous monitoring (Chen et al., 2023). Therefore, developing efficient and accurate remote sensing methods for estimating maize SPAD values has become essential for the sustainable development of maize production in the Yellow River irrigation area (Ma et al., 2024).

Unmanned aerial vehicle (UAV) remote sensing, with its high spatial and temporal resolution and operational flexibility, has become a mainstream approach for estimating crop physiological parameters (Gong et al., 2026). Meanwhile, smartphone cameras, which are portable, easy to operate, and low-cost, have gradually become useful complementary tools for rapid field monitoring (Li et al., 2023). If accurate cross-device calibration between UAV multispectral sensors and smartphone cameras can be achieved, the spectral, radiometric, and spatial-scale inconsistencies between the two observation systems can be reduced (Wang et al., 2024). Farmers can then photograph maize leaves in the field using an ordinary smartphone and a standardized procedure and, without any specialized knowledge or complex operations, rapidly obtain SPAD values and assess crop chlorophyll content and nutritional status, providing an immediate basis for field water and fertilizer regulation and stress management (Paul et al., 2025). However, the two devices differ substantially in sensor performance. The UAV multi-spectral camera provides broad band coverage and an accurate spectral response (Xia et al., 2022), whereas a smartphone camera has only three RGB bands, with inherently different sensitivity and spectral range. Moreover, UAV imagery represents canopy-level information containing leaves, shadows, and canopy gaps, whereas smartphone images mainly characterize individual leaves, resulting in an additional spatial-scale mismatch between the two devices. Consequently, SPAD estimation based directly on smartphone features is highly inaccurate (Speth et al., 2022). To address this problem, cross-device calibration has become an active research topic with the goal of establishing a high-accuracy mapping between heterogeneous sensors and eliminating spectral response differences. Current research mainly follows two directions: radiation correction and feature transfer. Some studies have used standard reference panels to construct physical conversion models for radiation correction. For example (Mohammadi et al., 2024), proposed a color-correction algorithm based on a 24-color standard color chart, establishing a mapping relationship between smartphone RGB values and UAV multispectral bands through polynomial regression, which effectively corrected deviations caused by changes in illumination and differences in sensor photosensitivity. Data-driven feature transfer methods have also shown considerable promise. For instance (Su et al., 2025), used machine learning algorithms to compensate for band gaps, constructing a conversion network from the RGB color space to the multispectral feature space, in which the high-accuracy UAV spectral information served as a “teacher” signal to guide feature learning from ground-based camera data. However, existing radiation correction methods have limited adaptability to complex field lighting, such as strong reflections or low-light conditions, and feature transfer models generally rely on large amounts of labeled data (Nuradili et al., 2024). Their stability under adverse conditions, such as drought stress typical of the Yellow River irrigation area, still requires further verification and optimization, which provides a key motivation for the present study.

This study focuses on UAV–smartphone calibration and SPAD estimation, with three main objectives: (1) to integrate spectral and texture features and select the optimal variables through correlation analysis, thereby overcoming the limited information provided by single features; (2) to compare three machine learning models, ridge regression (RR), support vector machine (SVM), and random forest (RF), to identify the best estimation model under arid stress conditions, addressing the lack of attention to stress adaptability in existing studies; and (3) to propose a smartphone-based background-extraction method and a cross-device calibration framework based on regularized least squares with a residual-compensation term, to improve feature consistency between the smartphone and the UAV and reconcile the competing demands of low-cost monitoring and high accuracy.

## 2 Materials and methods

### 2.1 Overview of the experimental site

**Figure 1:**
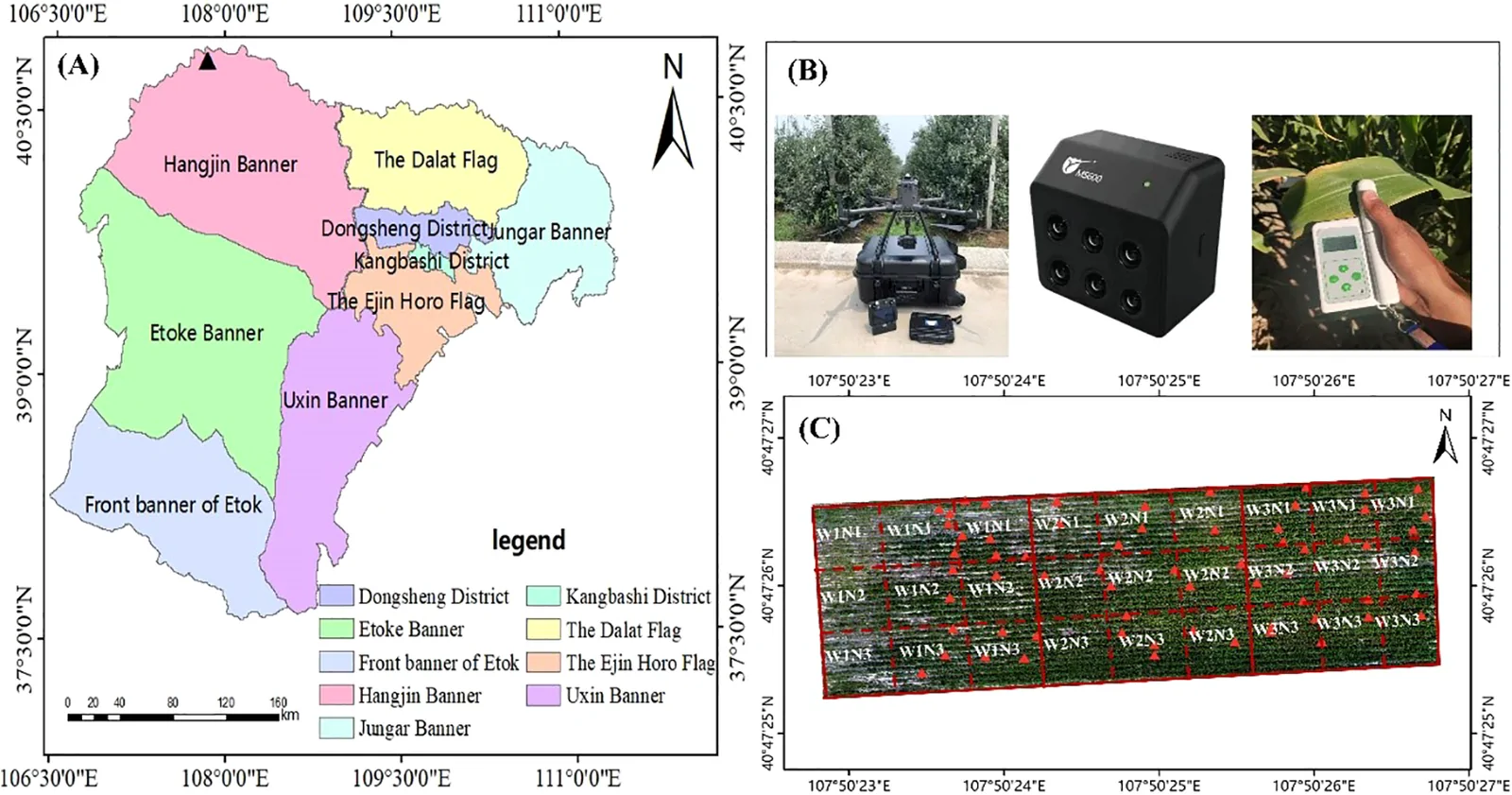
Location of the study area and layout of the experiments. This image is based on the map GS(2020)4619 from the standard map service website of the Ministry of Natural Resources. The boundary of the base map has not been modiﬁed. **(A)** Ordos City, Inner Mongolia. **(B)** Field sampling photos. **(C)** Layout of 27 plots with three irrigation levels (W1–W3) and three nitrogen rates (N1–N3), fully factorial with three replicates. Treatment combinations (e.g., W1N1, W2N1, …)are labeled within each plot and detailed in **Table 1**.

This experiment was conducted in 2023 in Guangmao Fifth Community, Jirigalangtu Town, northern Hangjin Banner, Ordos City, Inner Mongolia Autonomous Region, China (geographical coordinates: 107.9° E, 40.8° N). The location is shown in **Figure 1**, where the red triangles indicate the sampling points used in this study. The terrain is open and unobstructed and fully meets the requirements for stable UAV flights and large-scale observations. The regional climate is characterized by abundant sunlight and a large diurnal temperature range. Long-term meteorological records indicate a mean annual temperature of 7.9 °C and mean annual precipitation of only 168 mm. This arid climate with large temperature fluctuations has shaped the typical ecological and agricultural characteristics of the region.

The maize hybrid tested was the local cultivar “Ningyu 688”. Before sowing, the field was rotary-tilled and leveled. The experiment comprised nine treatments with three replicates each, for a total of 27 plots, each measuring 10 m × 4 m and arranged in a completely randomized design. Maize was cultivated according to local practices. Plastic film mulching was used with two rows per film; the film width was 60 cm, the wide and narrow row spacings were 1 m and 35 cm, respectively, and the plant spacing was 23 cm, giving a planting density of 61,100 plants ha^-^¹. Sowing was performed on May 29. The experiment used a two-factor factorial design combining irrigation and nitrogen fertilization. Three irrigation quotas were set at 50%, 75%, and 100% of the local irrigation amount (W1:150 m^3^/hm^2^, W2:225 m^3^/hm^2^, and W3:300 m^3^/hm^2^ per irrigation event). The crop was irrigated eight times during the growing season, with total irrigation amounts of 1200 m^3^/hm^2^ (W1), 1800 m^3^/hm^2^ (W2), and 2400 m^3^/hm^2^ (W3). Drip irrigation was used, and the applied volume was measured using water meters. Three nitrogen rates were applied as urea (N1:140 kg/hm^2^, N2:210 kg/hm^2^, and N3:280 kg/hm^2^). Nitrogen was top-dressed at the V6, V12, VT, and R3 stages in a ratio of 2:3:3:2, respectively. The basal fertilizer was a compound organic fertilizer (20% N, 25% P_2_O_5_, and 10% K_2_O), supplying 90 kg/hm^2^ of nitrogen. W3N3 represented the local farmers’ conventional irrigation and nitrogen regime. The details of the irrigation and fertilization treatments are presented in **Table 1**.

**Table 1:** Maize irrigation system.

Treatment	V6	V12	R3	Total irrigation volume	V6	V12	VT	R3	Total nitrogen application
				(m^3^/hm^2^)					(kg/hm^2^)
W1N1	300	600	300	1200	28	42	42	28	140
W1N2	300	600	300	1200	42	63	63	42	210
W1N3	300	600	300	1200	56	84	84	56	280
W2N1	450	900	450	1800	28	42	42	28	140
W2N2	450	900	450	1800	42	63	63	42	210
W2N3	450	900	450	1800	56	84	84	56	280
W3N1	600	1200	600	2400	28	42	42	28	140
W3N2	600	1200	600	2400	42	63	63	42	210
W3N3	600	1200	600	2400	56	84	84	56	280

V6, V12, VT, and R3 correspond to the jointing stage (June 10–20), flare opening stage (July 1–10), tasseling stage (July 20–30), and grain filling period (August 15–25), respectively. The sowing period is in early May.

### 2.2 Acquisition and processing of spectral images

#### 2.2.1 UAV multispectral imagery

UAV multispectral imagery was acquired using a DJI Matrice 300 RTK equipped with an MS600 Pro camera providing six bands: blue (B, 450 ± 35 nm), green (G, 555 ± 25 nm), red (R, 660 ± 20 nm), red-edge 1 (RE1, 720 ± 10 nm), red-edge 2 (RE2, 750 ± 15 nm), and near-infrared (NIR, 840 ± 35 nm). Images were collected from June to September 2023 at the main maize growth stages between 10:00 and 12:00 under clear conditions. Flights were conducted at 20 m altitude and 1.2 m s^-^¹ cruising speed with 80% forward and side overlap, and the sensor was oriented vertically downward. Black-and-white reference panels were used for radiometric calibration before each flight. 

**Figure 2:**
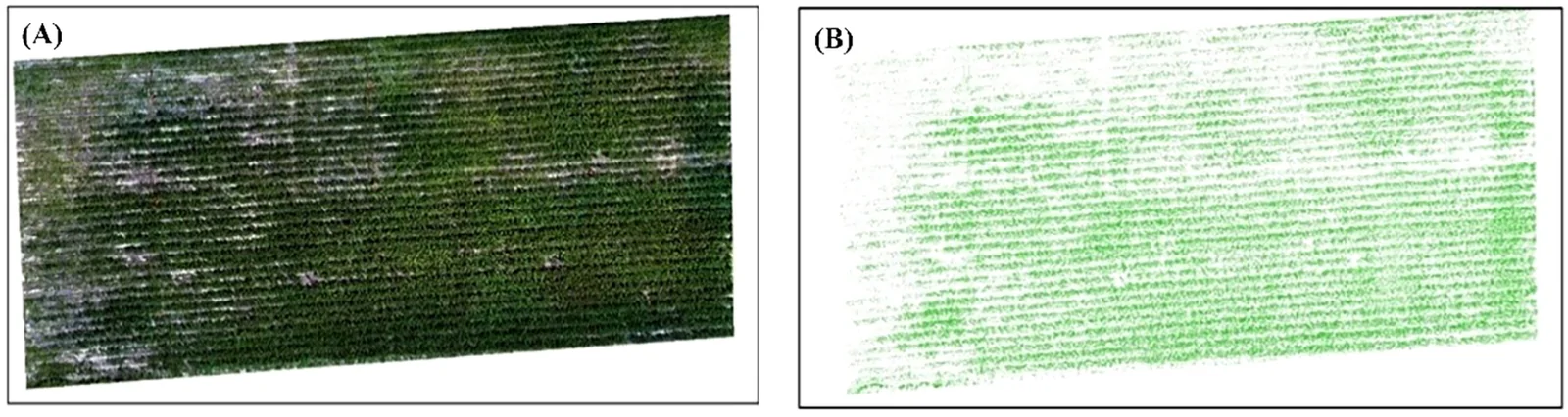
Comparison chart before and after removing soil background from spectral images. **(A)** Images of all maize pixels; **(B)** Images of maize pixels after soil background removal, taking the VT stage of maize as an example.

Raw multispectral images were processed in Pix4Dmapper to generate georeferenced orthomosaics, followed by band processing and feature extraction in ENVI (Environment for Visualizing Images). To reduce soil-background interference, a support vector machine (SVM) classifier trained with 30 maize and 30 soil samples was used to generate a maize mask (**Figure 2**). Classification accuracy exceeded 98%, with Kappa coefficients above 0.96 (**Table 2**). All subsequent UAV feature extraction was performed on the masked maize canopy images.

**Table 2:** Classification accuracy and Kappa coefficient of support vector machine.

Childbearing period	Overall accuracy (%)	Kappa coefficient
V6	99.21	0.982
VT	98.35	0.963
R3	98.11	0.972

#### 2.2.2 Smartphone RGB imagery

Smartphone RGB images were acquired using an iPhone 13 Pro Max immediately after UAV acquisition under clear field conditions. Automatic white balance and exposure were disabled, the focal length was fixed, and the camera was positioned perpendicular to the target leaf at a distance of 30–40 cm. Each image contained a single functional leaf occupying more than 70% of the frame, while backlighting, severe occlusion, and strong specular reflection were avoided. The images were stored as 8-bit RGB data, representing the digital intensity of leaf pixels.

**Figure 3:**
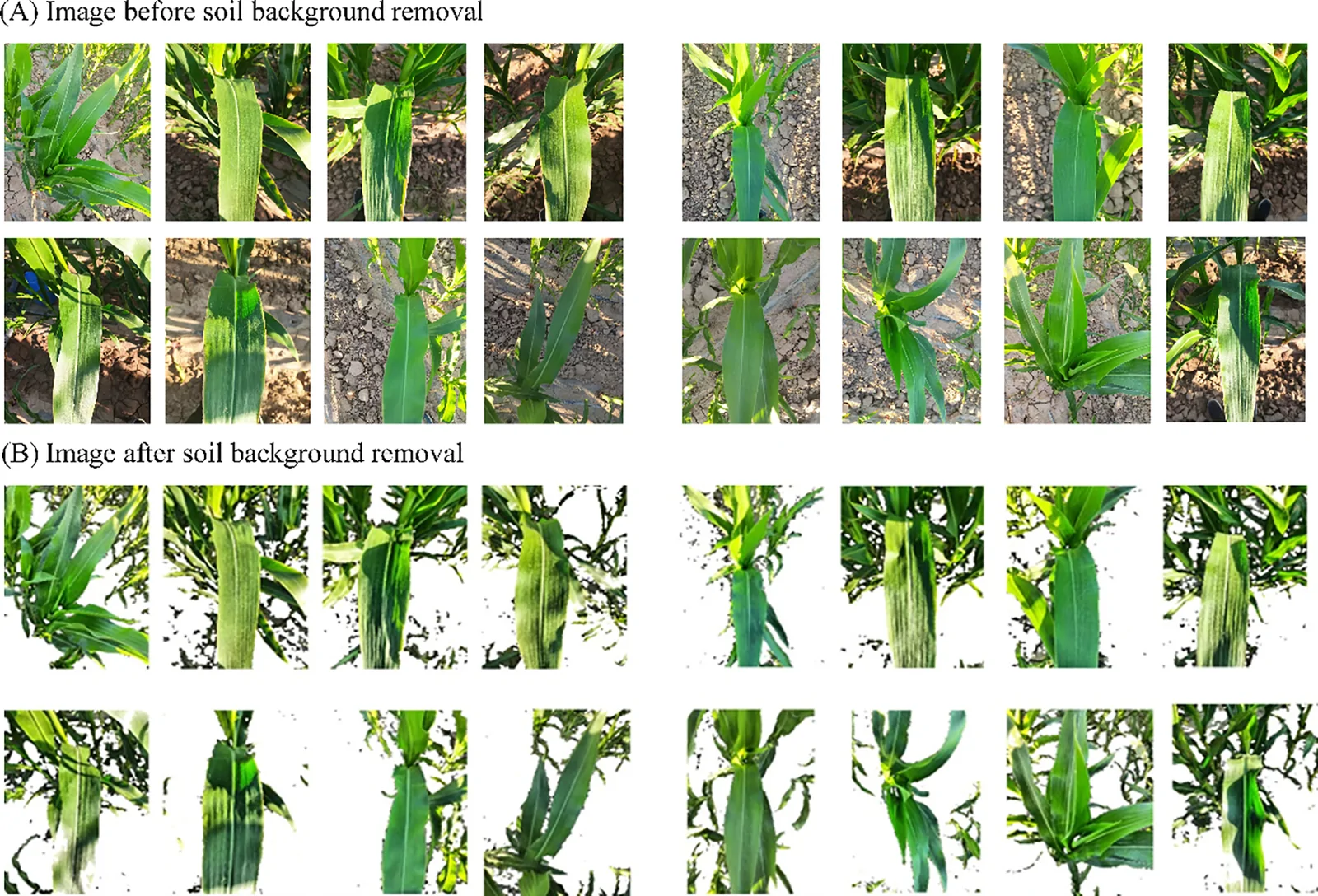
Schematic illustration of smartphone images before and after soil background removal. **(A)** Original image; **(B)** Image after soil background removal using HSV‑based green‑color segmentation. This representative example was taken at the V6 stage from a plot under the W2N2 treatment. The same procedure was applied consistently to all images across all growth stages and treatments.

Leaf-background separation was performed in HSV (Hue, Saturation, Value) color space using thresholds of H = 30–85°, S = 35–255, and V = 30–255. The mask was refined using 7 × 7 morphological opening and closing operations, followed by median filtering and CLAHE-based illumination adjustment. Images were then standardized to 960 × 1280 pixels, and only the segmented leaf region was retained for subsequent spectral and texture feature extraction, UAV–smartphone calibration, and SPAD estimation (**Figure 3**).

### 2.3 Acquisition of ground measured data

Ground SPAD measurements were conducted nondestructively using a calibrated SPAD-502 chlorophyll meter immediately after UAV and smartphone image acquisition at each growth stage. Fifteen representative maize plants were selected from each plot, avoiding plants affected by disease, pests, or mechanical damage. For each plant, a representative functional leaf was selected, and SPAD values were measured at the upper, middle, and lower positions using a calibrated SPAD-502 chlorophyll meter. Each position was measured three times, and the mean value after removing outliers was used as the representative SPAD value. The geographic coordinates and plant identifiers of all sampled plants were recorded.

Cross-device matching was performed at the plant level rather than by direct pixel-to-pixel correspondence. UAV orthomosaics were aligned with the sampling coordinates, and the mean spectral values within a 3 × 3 pixel neighborhood centered on each sampling position were extracted. Smartphone images were matched with the corresponding UAV observations and SPAD measurements using acquisition time, GPS coordinates, and plant identifiers. Thus, UAV canopy-level features, smartphone leaf-level features, and ground SPAD measurements were paired for subsequent cross-device calibration.

### 2.4 Spectral and texture feature extraction

For UAV imagery, six multispectral bands (B, G, R, RE1, RE2, and NIR) and six SPAD-related spectral indices were used, resulting in 12 spectral variables. For smartphone imagery, RGB bands and corresponding visible-band indices were calculated as listed in **Table 3**. Spectral variables were extracted from the matched sampling locations in ENVI.

**Table 3:** Calculation of spectral parameters.

Equipment	Spectral parameter (SI)	Calculation formula	Document serial number
UAV	Single band	B, G, R, RE1, RE2, NIR	
	DVI	(NIR-R)	(Naqvi et al., 2021)
	NLI	(NIR^2-R)/(NIR^2 +R)	(Huang et al., 2023)
	RDVI	(NIR-R)/(NIR+R)^0.5	(Yang and Du, 2021)
	GNDVI	(NIR-G)/(NIR+G)	(Zhang et al., 2019)
	NDVI	(NIR-R)/(NIR+R)	(Gracia-Romero et al., 2018)
Mob	Single band	R, G, B	
	NGRDI	(G-R)/(G+R)	(Zheng et al., 2018)
	VARI	(G-R)/(G+R-B)	(Emard, 2024)
	EXG	2G-R-B	(Chen et al., 2025)
	RCRI	G/R	(Colovic et al., 2024)

For the multi-spectral image, the single bands R, G, B, RE1, RE2, and NIR represent red, green, blue, red edge 1, red edge 2, and near-infrared respectively. Among them, DVI: Difference Vegetation Index; NLI: Nonlinear Vegetation Index; RDVI: Re-normalized Vegetation Index; GNDVI: Green Normalized Difference Vegetation Index; NDVI: Normalized Vegetation Index; NGRDI: Normalized Green-Red Difference Index; VARI: Visible Light Atmospheric Attenuation Index; EXG: Visible Light Vegetation Index; RCRI: Green-Red Ratio Index.

Texture features were derived from each UAV and smartphone band using the grey-level co-occurrence matrix (GLCM) in ENVI 5.6. Eight texture features were calculated: mean, variance, homogeneity, contrast, dissimilarity, entropy, angular second moment, and correlation. A 7 × 7 moving window with pixel offsets of 1 in both directions was used. Spectral indices (SI) and texture features (TI) were subsequently screened according to their relationships with measured SPAD values (Zhang et al., 2026).

### 2.5 UAV–smartphone cross-device calibration

#### 2.5.1 Core idea

Because smartphones provide only three bands (R, G, and B), they cannot directly capture red-edge and near-infrared information that is sensitive to SPAD. In this study, a mathematical mapping between the smartphone feature space and UAV multispectral feature space was established to compensate for this missing information. The aim of this transfer was to use the readily obtainable smartphone RGB and texture features to simulate the UAV multispectral response through a mapping matrix, and then to input the simulated features into the pre-trained optimal UAV SPAD model, thereby achieving high-accuracy SPAD estimation from smartphone images (Heikkinen et al., 2007).

#### 2.5.2 Mathematical model

Let the filtered smartphone feature vector be ${\text{X}}_{mob}={\left[x_{mob1},x_{mob2},\ldots ,x_{mobM}\right]}^{T}$ (M- dimensional) and the filtered UAV feature vector be ${\text{X}}_{uav}={\left[x_{uav1},x_{uav2},\ldots ,x_{uavN}\right]}^{T}$ (N- dimensional).

To incorporate the bias term (residual compensation) into the matrix operations, the extended smartphone feature vector was defined as ${\tilde{X}}_{mob}={\left[X_{mob}^{T},1\right]}^{T}$. The equivalent UAV features were then obtained as follows in Equation 1:

(1) $${\hat{X}}_{uav}=W\cdot {\tilde{X}}_{mob}$$

#### 2.5.3 Solution of the migration matrix:

A training set was constructed from paired samples (smartphone and UAV features of the same plant at the same time). The mapping matrix W was solved using regularized least squares (ridge regression), with the objective function presented in Equation 2:

(2) $$\min\left({//X_{uav}-\left(K\cdot X_{mob}+\epsilon \right)//}_{2}^{2}+\lambda {//W//}_{F}^{2}\right)$$

Where λ is the regularization parameter selected by cross-validation to prevent overfitting. Once W is solved, any newly acquired smartphone features can be mapped to the equivalent UAV features and then input directly into the UAV prediction model (Liu et al., 2025).

To determine the optimal regularization parameter λ, 10-fold cross-validation combined with a grid search was used for hyperparameter tuning. Candidate λ values were generated on a logarithmic scale from 10^–4^ to 10^2^ with 50 equally spaced intervals. For each candidate, the mean cross-validation root-mean-square error (RMSE) within the training set was computed, and the λ yielding the minimum RMSE was selected. The procedure yielded optimal λ values of 0.015, 0.008, and 0.021 for the jointing, tasseling, and grain-filling stages, respectively. This strategy balances search efficiency and accuracy while ensuring the generalization ability of the calibration matrix.

### 2.6 Specific model methods

Three machine-learning regression models, ridge regression (RR), support vector machine (SVM), and random forest (RF), were compared for maize SPAD estimation under different feature combinations and growth stages. These models were selected because they represent different modeling strategies. RR is a linear regression method with L2 regularization that reduces coefficient instability and limits overfitting in the presence of correlated predictors. SVM performs nonlinear regression by mapping input features into a higher-dimensional feature space through kernel functions and is suitable for relatively small datasets with nonlinear relationships. RF is an ensemble-learning method that integrates multiple decision trees through bootstrap sampling and random feature selection, enabling it to capture complex nonlinear relationships while reducing the risk of overfitting.

For model development and evaluation, the complete dataset was divided into a training set (70%) and a validation set (30%) using a plot-based stratified sampling strategy. Model construction and prediction were implemented in Python using the scikit-learn library, and the resulting model performance was visualized in Origin 2021.

### 2.7 Model evaluation metrics

The model accuracy was evaluated using the coefficient of determination (R^2^) and RMSE, as shown in Equations 3 and 4, respectively. A higher R^2^ and lower RMSE indicate better model performance. These metrics were also used to evaluate the accuracy of the machine learning models and UAV–smartphone calibration.

(3) $$R^{2}=\frac{{\left[\sum_{i=1}^{n}\left(X_{i}-\bar{X}\right)\left(Y_{i}-\bar{Y}\right)\right]}^{2}}{\sum_{i=1}^{n}{\left(X_{i}-\bar{X}\right)}^{2}\sum_{i=1}^{n}{\left(Y_{i}-\bar{Y}\right)}^{2}}$$

(4) $$RMSE=\sqrt{\frac{\sum_{i=1}^{n}{\left(Y_{i}-\bar{Y}\right)}^{2}}{n}}$$

where *X_i_*, $\bar{X}$, *Y_i_*, and $\bar{Y}$ denote the measured value, the mean measured value, the estimated value, and the mean estimated value, respectively, and n is the number of samples.

### 2.8 Statistical analysis

All SPAD measurements were analyzed using a two-way analysis of variance (ANOVA) to evaluate the effects of irrigation regime (W), nitrogen application rate (N), and their interaction (W × N) on maize SPAD values at each growth stage. The significance of differences among treatment means was tested using Duncan’s multiple range test at a significance level of P< 0.05. All statistical analyses were performed using SPSS Statistics (version 26.0, IBM Corp., Armonk, NY, USA). Results are presented as mean ± standard deviation (SD).

## 3 Results and analysis

### 3.1 Effects of different treatments on SPAD values of maize

At the V6 stage, SPAD values differed markedly among treatments. The W3N3 treatment had the highest SPAD value and W1N1 the lowest, because the V6 stage is a critical period for maize growth, and low water and low fertilizer conditions restrict maize growth. At the VT stage, under the W2 irrigation level, SPAD was 2.7% higher under high nitrogen than under medium nitrogen and 6.9% higher under low nitrogen. Under the N2 condition, SPAD under high water was 6.1% lower than low water but 22.1% higher than under low water. At the grain-filling stage, SPAD reached its peak; under the W2 and W1 levels, SPAD values were higher under W3, with no significant difference between W2 and W1. At maturity, the leaves turned yellow and SPAD declined rapidly, with no clear pattern among treatments (**Table 4**); therefore, this stage was excluded from the subsequent modeling.

**Table 4:** Effects of interaction of water and nitrogen on maize chlorophyll (SPAD) at different growth stages.

Irrigation treatment	Fertilization treatment	V6	VT	R3
W1	N1	25.24 ± 1.98 e	30.22 ± 2.93cd	36.71 ± 2.29a
	N2	28.18 ± 2.77 cde	30.12 ± 1.31d	35.11 ± 5.5ab
	N3	26.06 ± 1.67de	30.6 ± 3.91bcd	37.97 ± 4.07a
W2	N1	27.02 ± 1.83de	36.18 ± 3.47a	34.53 ± 6.36ab
	N2	30.69 ± 2.19bc	37.79 ± 1.96a	38.32 ± 6.75a
	N3	28.62 ± 1.05bcd	38.7 ± 2.05a	30.54 ± 4.85b
W3	N1	31.52 ± 1.58b	35.4 ± 3.37abc	31.16 ± 3.06b
	N2	34.88 ± 1.63a	36.87 ± 2.91a	32.69 ± 2.89b
	N3	37.06 ± 1.36a	35.47 ± 2.77ab	30.24 ± 3.39b

Values are presented as mean ± standard deviation (SD). Different lowercase letters within the same column indicate significant differences among treatments at the *P*< 0.05 level, as determined by two-way ANOVA followed by Duncan’s multiple range test. W and N represent irrigation regime and nitrogen application rate, respectively. The same below.

### 3.2 Selection based on spectral indices of drones and cameras

**Figure 4:**
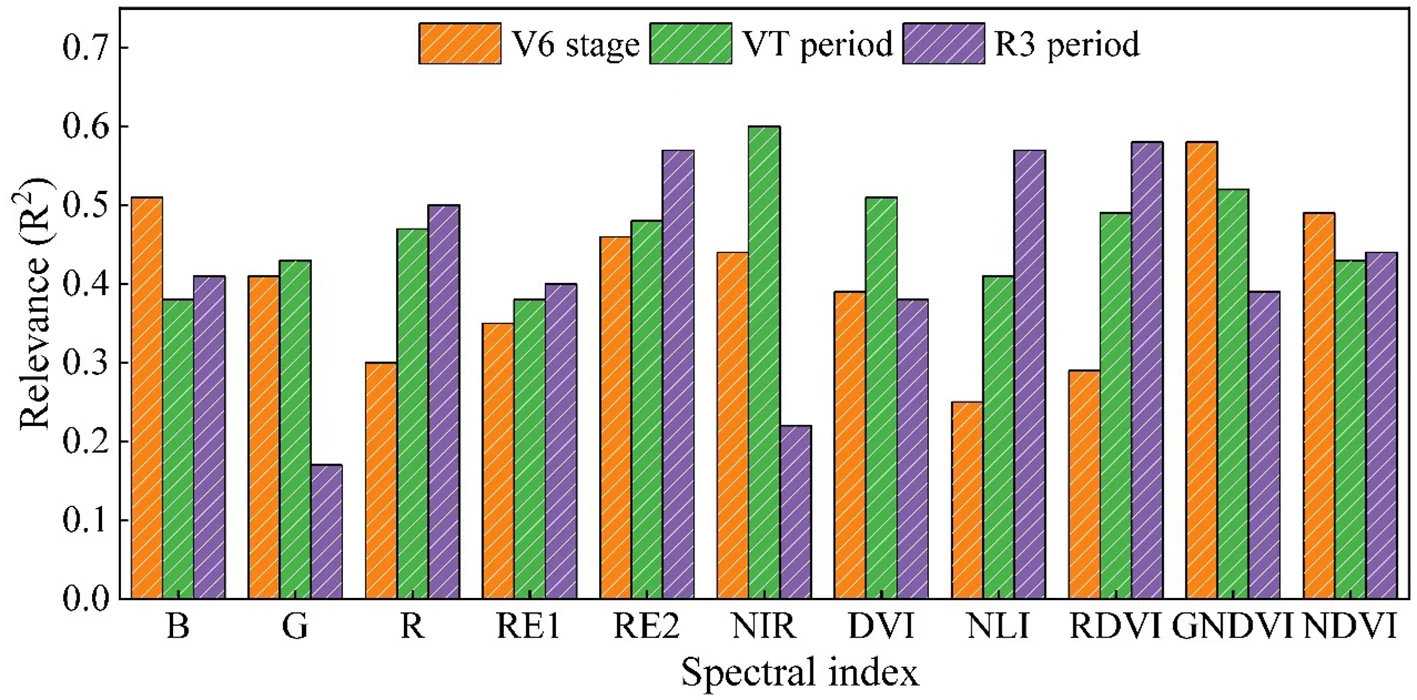
Correlation of unmanned aerial vehicle spectral indices. B, blue band; G, green band; R, red band; RE1, red-edge 1 band, 720 ± 10 nm; RE2, red-edge 2 band, 750 ± 15 nm; NIR, near-infrared band, 840 ± 35 nm; DVI, Difference Vegetation Index; NLI, Nonlinear Vegetation Index; RDVI, Re-normalized Difference Vegetation Index; GNDVI, Green Normalized Difference Vegetation Index; NDVI, Normalized Difference Vegetation.

Based on UAV imagery, vegetation indices were calculated from six single-band reflectances (B, G, R, RE1, RE2, and NIR) to construct the final spectral variables. Correlation analysis was then performed between the measured maize SPAD values at each stage and the UAV-derived spectral variables, and the four most highly correlated indices were selected for each stage. At the V6 stage, all spectral variables had correlations greater than 0.3; among them, B, RE2, GNDVI, and NDVI had absolute correlations greater than 0.4 (**Figure 4**). At the VT stage, reflectance was strongly correlated with SPAD, and NIR, DVI, RDVI, and GNDVI showed relatively high correlation, all approximately 0.5. At the grain-filling stage, four indices, R, RE2, NLI, and RDVI, were selected, all of which had correlations greater than 0.4.

**Figure 5:**
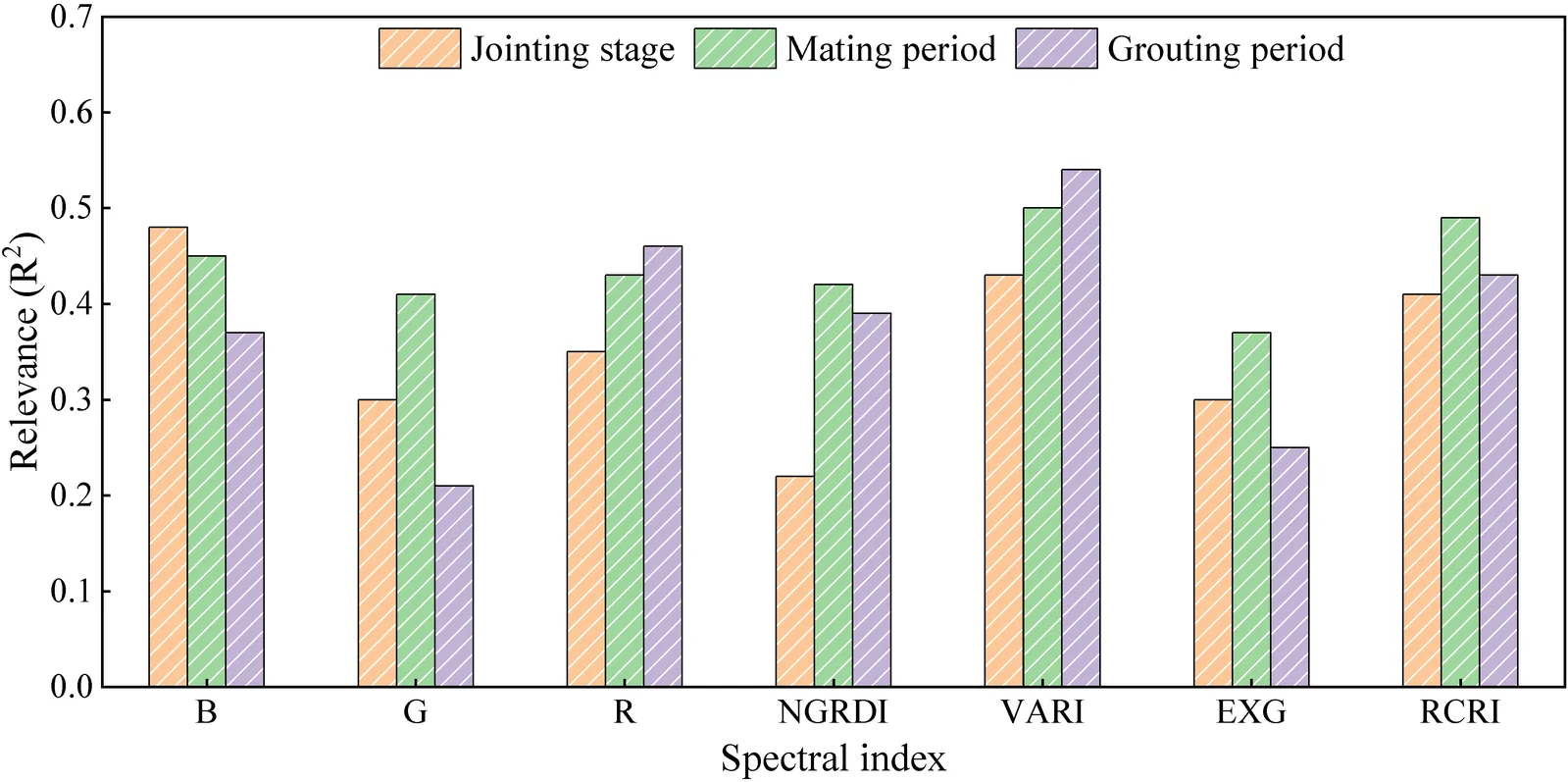
Correlation of spectral indexes of the camera. B, blue band; G, green band; R, red band; NGRDI, Normalized Green-Red Difference Index; VARI, Visible Atmospherically Resistant Index; EXG, Excess Green Index; RCRI, Red-Green Ratio Index.

Based on the smartphone images, vegetation indices were calculated from the three single-band reflectances (B, G, and R) to construct the final spectral variable. Correlation analysis was also performed between the measured SPAD values at each stage and the smartphone spectral variables, and the four most highly correlated indices were selected. At the V6 stage, all spectral variables had correlations greater than 0.2; among them, B, R, VADI, and RCRI had absolute correlations greater than 0.3 (**Figure 5**). At the VT stage, reflectance was strongly correlated with SPAD, with all spectral indices being greater than 0.4, and B, G, R, and VARI were selected as the final spectral variables. At the grain-filling stage, four indices, R, NGRDI, VARI, and RCRI, were selected, all of which had correlations greater than 0.4.

### 3.3 Texture feature selection for the UAV and the smartphone

**Figure 6:**
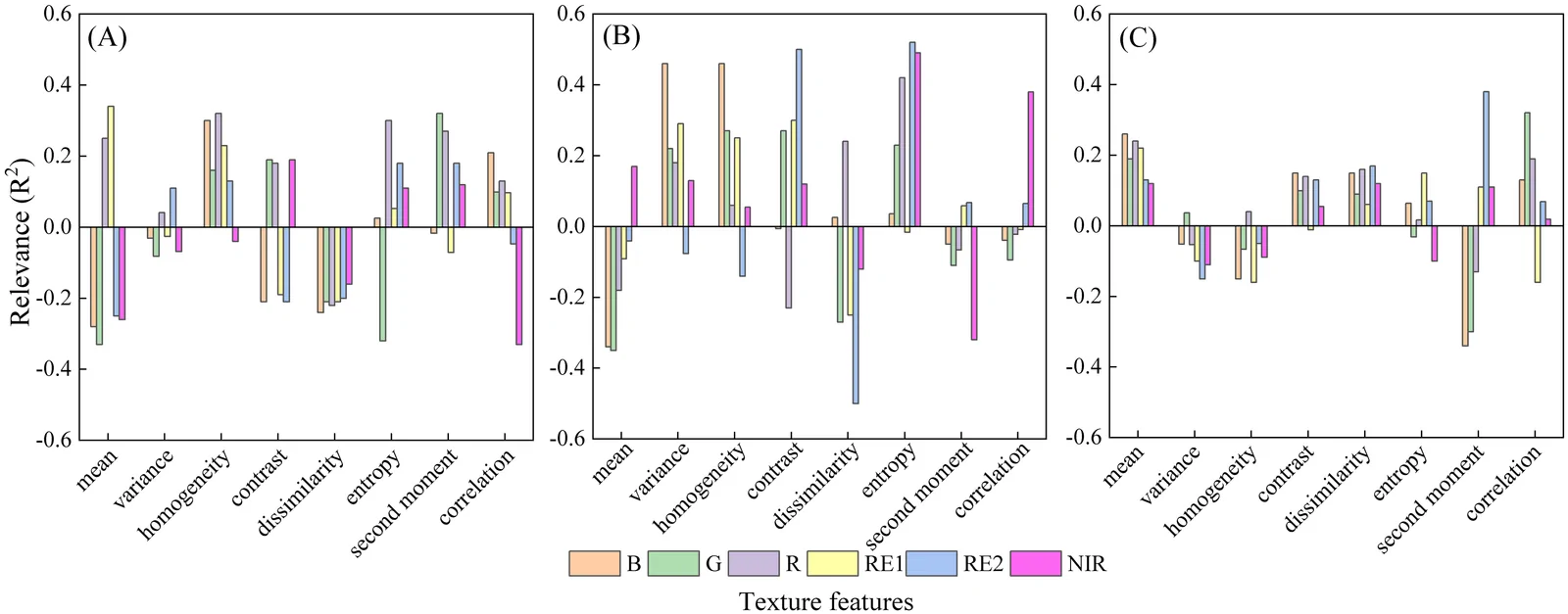
Correlation Analysis of Texture Features and SPAD. **(A)** V6 stage; **(B)** VT stage; **(C)** R3 stage. Mean, variance, homogeneity, contrast, dissimilarity, entropy, second moment, and correlation are texture statistics derived from the grey-level co-occurrence matrix (GLCM).

Based on the UAV images, six single-band texture sets (B, G, R, RE1, RE2, and NIR) were extracted, with each band yielding eight texture features. Correlation analysis was performed between the measured maize SPAD values at each stage and the UAV-derived texture features, and the two most highly correlated features were selected. At the V6 stage, the correlation between the texture features and SPAD was weak, with a maximum of approximately 0.3 (**Figure 6A**); RE1-mean and G-second moment, which had relatively higher correlations, were therefore selected. At the VT stage, the correlation was strong, with some features reaching approximately 0.5, and RE2-entropy and RE2-contrast were selected as the optimal combination (**Figure 6B**). At the R3 stage, the correlation was even weaker, and the NIR second moment and G-correlation were selected as the optimal combination (**Figure 6C**).

**Figure 7:**
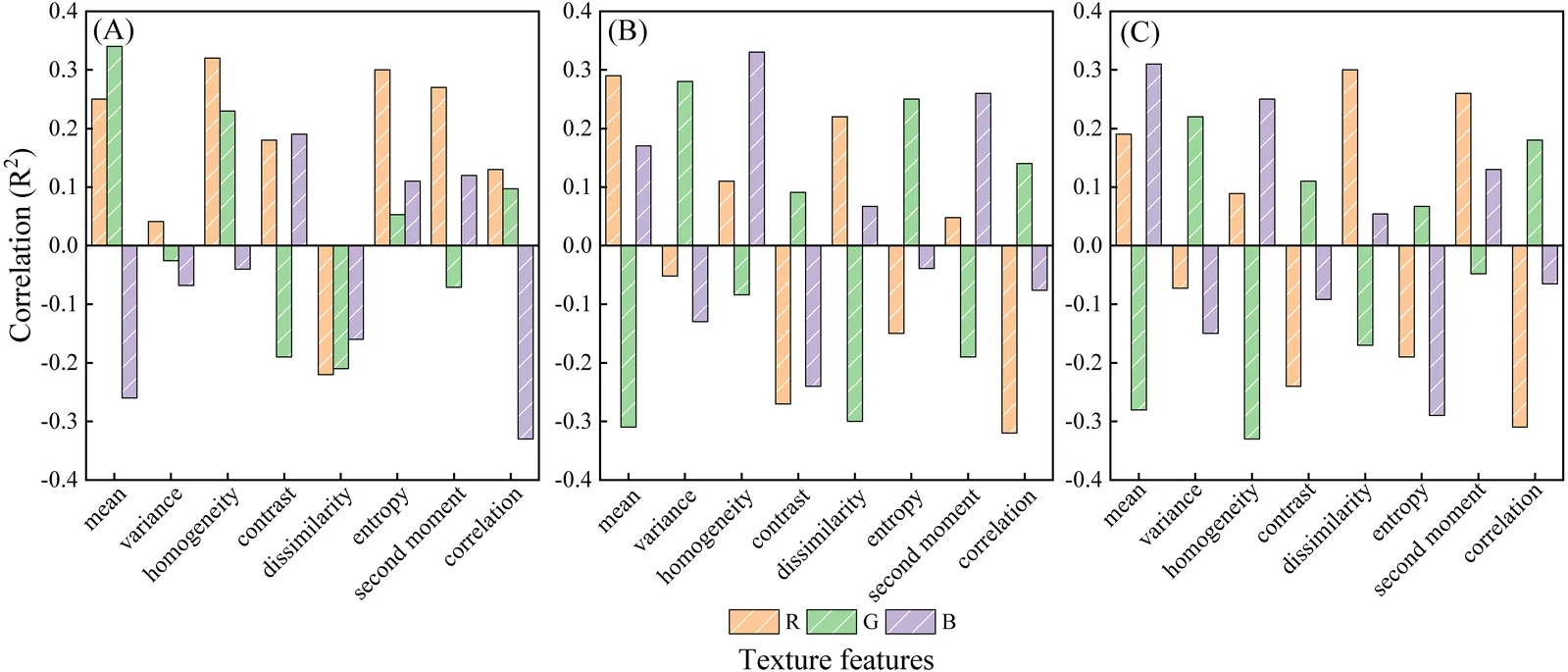
Correlation Analysis of Texture Features and SPAD. **(A)** V6 stage; **(B)** VT stage; **(C)** R3 stage. Mean, variance, homogeneity, contrast, dissimilarity, entropy, second moment, and correlation are texture statistics derived from the grey-level co-occurrence matrix (GLCM).

Based on the smartphone images, three single-band texture sets (B, G, and R) were extracted, with each band yielding eight texture features. A correlation analysis was performed between the measured SPAD values at each stage and the smartphone-derived texture features, and the two most highly correlated features were selected. At the V6 stage, the correlation was relatively clear at approximately 0.3 (**Figure 7A**), and G-mean and R-homogeneity were selected. At the VT stage, B-homogeneity and G-variance were selected as the optimal combinations (**Figure 7B**). At the R3 stage, B-mean and R-dissimilarity were selected as the optimal combination (**Figure 7C**).

### 3.4 UAV multi-feature estimation model

**Figure 8:**
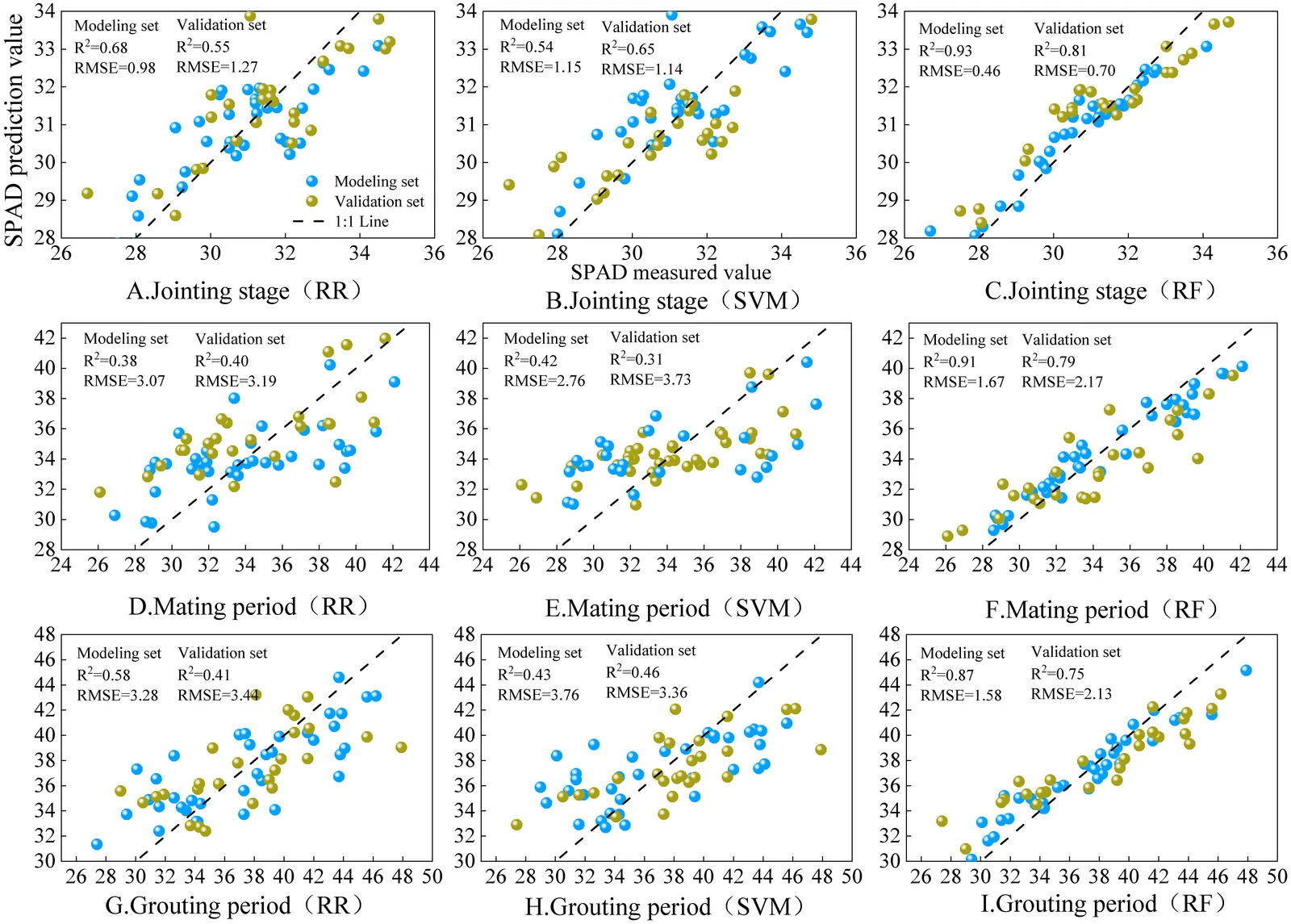
Comparison of SPAD estimation performance using three machine learning models (RR, SVM, and RF) based on combined spectral and texture features (SI + TI) from UAV multispectral imagery at different growth stages. **(A–C)** V6 stage; **(D–F)** VT stage; **(G–I)** R3 stage. For each model, the scatter plots show the relationship between measured and estimated SPAD values, with the modeling set (calibration) and validation set presented together. The solid line represents the 1:1 reference line. RR, Ridge Regression; SVM, Support Vector Machine; RF, Random Forest; R², coefficient of determination; RMSE, root mean square error; V6, sixth-leaf stage, jointing; VT, tasseling stage; R3, grain-filling stage; SI, spectral indices; TI, texture features.

At each growth stage, the four spectral features and two texture features of the UAV were combined into different variable sets (**Table 5**), and SPAD values were estimated using RR, RF, and SVM models (**Figure 8**). The combination of remote sensing features improved the accuracy of the SPAD estimation models. At the V6 stage, the RF model performed best, with a training-set R^2^ of 0.93 and RMSE of 0.46, 26% and 41% higher than the RR and SVM models, respectively, and a validation-set R^2^ of 0.81 and RMSE of 0.70, which were 16% and 33% higher, respectively (**Figures 8A-C**). At the VT stage, the RF training-set R^2^ reached 0.91, 7.1% and 9.6% higher than RR and SVM, and the validation-set R^2^ was 0.79, 27.4% and 41% higher, respectively (**Figures 8D–F**). At the R3 stage, the RF training-set R² reached 0.87, which was 2.4% and 12.9% higher than those of RR and SVM, respectively, and the validation-set R² was 0.75, which was 19% and 21% higher, respectively (**Figures 8G–I**). Overall, the RF model built from the combined remote sensing features achieved markedly higher accuracy and estimated maize SPAD values well at all growth stages.

**Table 5:** Final screening results of feature variables.

Equipment	Period	Type	Quantity	Filtering results
UAV	V6	(SI)	4	B, RE2, GNDVI, NDVI
		(TI)	2	RE1-mean, G-second moment
		(SI+TI)	6	B, RE2, GNDVI, NDVI, RE1-mean, G-second moment
	VT	(SI)	4	NIR, DVI, RDVI, GNDVI
		(TI)	2	RE2-entropy, RE2-contrast
		(SI+TI)	6	NIR, DVI, RDVI, GNDVI, RE2-entropy, RE2-contrast
	R3	(SI)	4	R, RE2, NLI, RDVI
		(TI)	2	NIR-second moment, G-correlation
		(SI+TI)	6	R, RE2, NLI, RDVI, NIR-second moment, G-correlation
Mob	V6	(SI)	4	B, R, VADI, RCRI
		(TI)	2	G-mean, R-homogeneity
		(SI+TI)	6	B, R, VADI, RCRI, G-mean, R-homogeneity
	VT	(SI)	4	B, G, R, VARI
		(TI)	2	B-homogeneity, G-variance
		(SI+TI)	6	B, G, R, VARI, B-homogeneity, G-variance
	R3	(SI)	4	R, NGRDI, VARI, RCRI
		(TI)	2	B-mean, R-dissmilarity
		(SI+TI)	6	R, NGRDI, VARI, RCRI, B-mean, R-dissmilarity

UAV, unmanned aerial vehicle; Mob, smartphone; SI, spectral indices; TI, texture features; V6, sixth-leaf stage (jointing); VT, tasseling stage; R3, milk stage (grain-filling period); B, G, R, RE1, RE2, and NIR represent the blue, green, red, red-edge 1, red-edge 2, and near-infrared bands, respectively; GNDVI, Green Normalized Difference Vegetation Index; NDVI, Normalized Difference Vegetation Index; DVI, Difference Vegetation Index; RDVI, Re-normalized Difference Vegetation Index; NLI, Nonlinear Vegetation Index; VARI, Visible Atmospherically Resistant Index; NGRDI, Normalized Green-Red Difference Index; RCRI, Red-Green Ratio Index; mean, entropy, contrast, second moment, homogeneity, variance, and dissimilarity are texture statistics derived from the grey-level co-occurrence matrix (GLCM).

### 3.5 Calibration and SPAD estimation from multiple smartphone features

**Figure 9:**
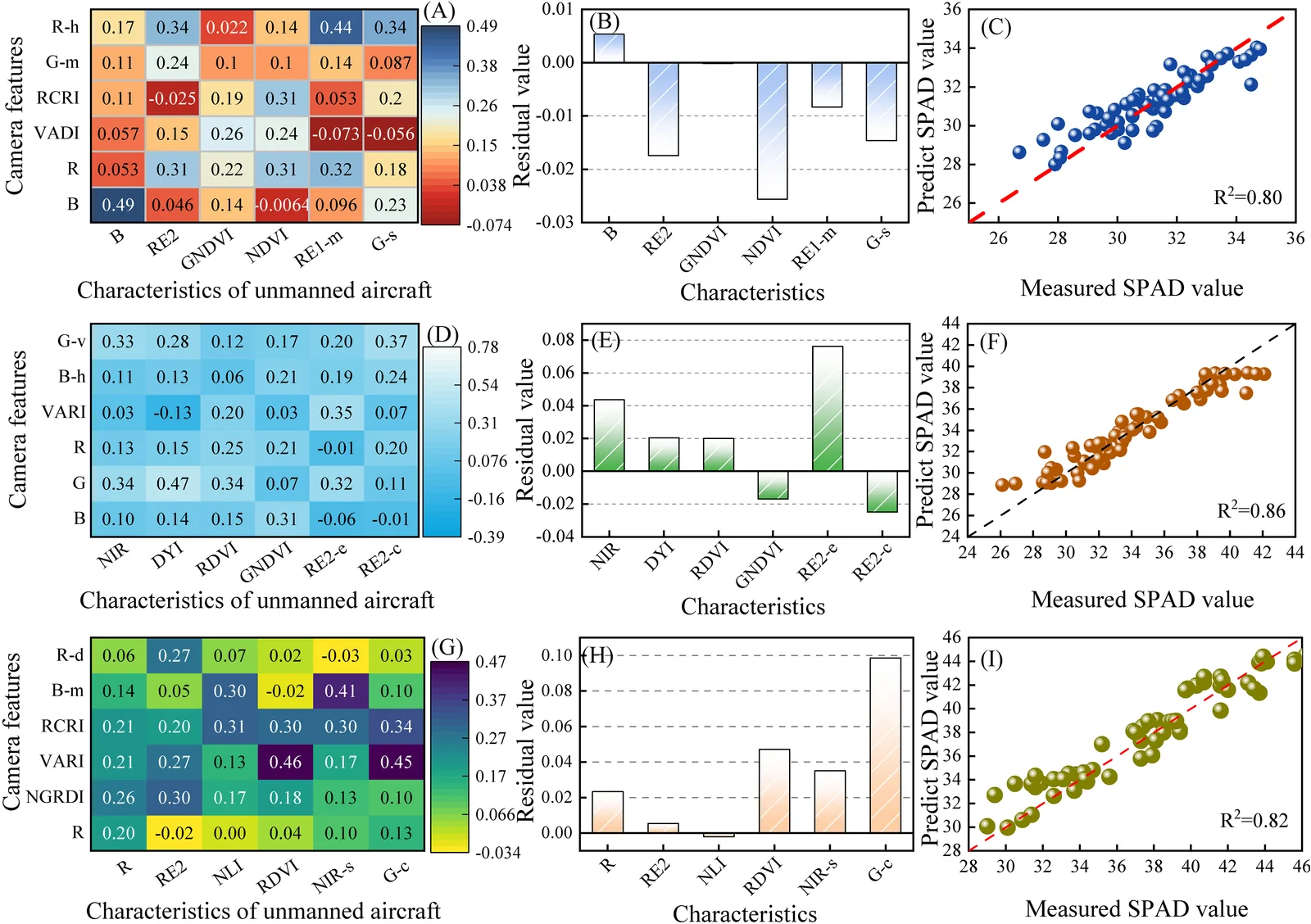
Camera calibration and prediction graph. **(A–C)** represent the V6 stage, **(D–F)** represent the VT stage, and **(G–I)** represent the R3 stage. The first row shows the calibration coefficient between camera features and unmanned aerial vehicle features. The second column represents the residual distribution of the mapping of booting period features. The third column represents the inversion accuracy of the calibrated model.

To ensure the independence of the calibrated input features, variance inflation factor (VIF) diagnostics were performed. All VIF values were below 5.0, effectively avoiding multicollinearity issues. In the calibration coefficient matrix K obtained by regularized least squares, highly correlated physical features received higher mapping weights, whereas the weights of weakly correlated features approached zero. For example, at the jointing stage (**Figure 9A**), the calibration coefficient between smartphone B and UAV B reached 0.48, whereas that between smartphone B and UAV NDVI was only -0.0064; at the tasseling stage (**Figure 9D**), the coefficient between smartphone G and UAV DVI reached 0.47; and at the grain-filling stage (**Figure 9G**), the coefficient between smartphone VARI and UAV RDVI reached 0.46. The median residual remained stable between -0.04 and 0.1, except for the RE2-entropy residual at tasseling (**Figure 9E**) and the RE2-contrast residual at grain filling (**Figure 9H**), which approached 0.1, confirming the robustness of the feature mapping. In terms of mapping accuracy (**Table 6**), the cross-device consistency of the spectral features (R² = 0.85–0.92, RMSE = 0.018–0.025) was considerably better than that of the texture features (R² = 0.72–0.81, RMSE = 0.029–0.038). Finally, the calibrated equivalent features were input into the optimal RF model for SPAD estimation, which markedly improved accuracy and yielded a reasonable value distribution: at jointing (**Figure 9C**), the predicted range was 26–36 with R² = 0.80; at tasseling (**Figure 9F**), performance was best, with a predicted range of 26–40 and R² = 0.86; and at grain-filling (**Figure 9I**), the predicted range was 30–44 with R² = 0.82. These results indicate that feature calibration effectively corrected the physical differences between the sensors, enabling a high-accuracy field SPAD estimation from smartphone images.

**Table 6:** Evaluation of calibration feature mapping accuracy.

Period	Type	RMSE (feature mapping)	R² (feature mapping)
V6	(SI)	0.021	0.89
	(TI)	0.034	0.76
VT	(SI)	0.018	0.92
	(TI)	0.029	0.81
R3	(SI)	0.025	0.85
	(TI)	0.038	0.72

V6, sixth-leaf stage (jointing); VT, tasseling stage; R3, milk stage (grain-filling period); SI, spectral indices; TI, texture features; RMSE, root mean square error; R², coefficient of determination.

### 3.6 Inversion of maize SPAD values

**Figure 10:**
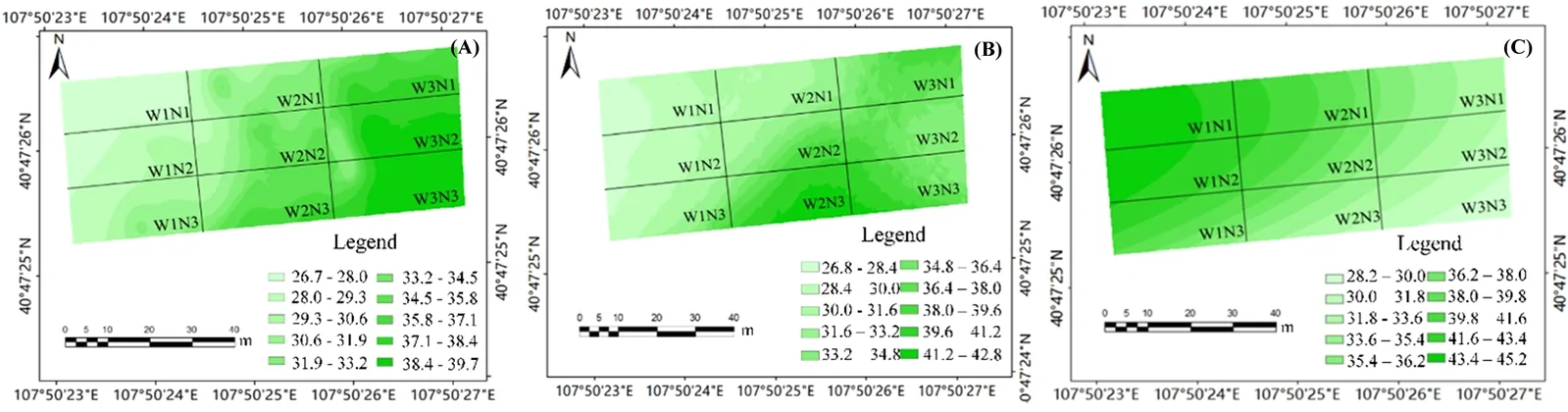
Spatial distribution of maize SPAD values estimated by the optimal RF model at the field scale across different growth stages. **(A)** V6 stage (jointing); **(B)** VT stage (tasseling); **(C)** R3 stage (grain-filling). The color bars indicate the predicted SPAD values for each plot.

At the jointing stage, the high-water-high-nitrogen (W3N3) plots formed a distinct high-value zone with maize SPAD values above 37, whereas the low-water-low-nitrogen (W1N1) plots formed a low-value zone with SPAD values below 26. This spatial pattern agreed well with the field water and nitrogen gradients (**Figure 10A**). At the tasseling stage (**Figure 10B**), the medium-water-high-nitrogen (W2N3) plots reached SPAD values of 39–42, which was the highest of all treatments. Under high water (W3), the SPAD values were lower than those under the corresponding W2 treatments, indicating that moderate water stress promoted chlorophyll accumulation. At the grain-filling stage (**Figure 10C**), SPAD values under the low- and medium-water treatments (W1, W2) were higher than under high water (W3), with W2N2 (38.32) and W1N3 (37.97) the most prominent, whereas the W3N3 treatment formed a clear low-value zone (around 30), consistent with the field measurements. A comparison of the estimation maps with the actual field layout showed only small variations among replicate plots, and the spatial patterns of the water-N interaction matched the observed statistical trends, confirming the strong spatial consistency of the estimation results and their usefulness for precise field-scale water and fertilizer management.

**Figure 11:**
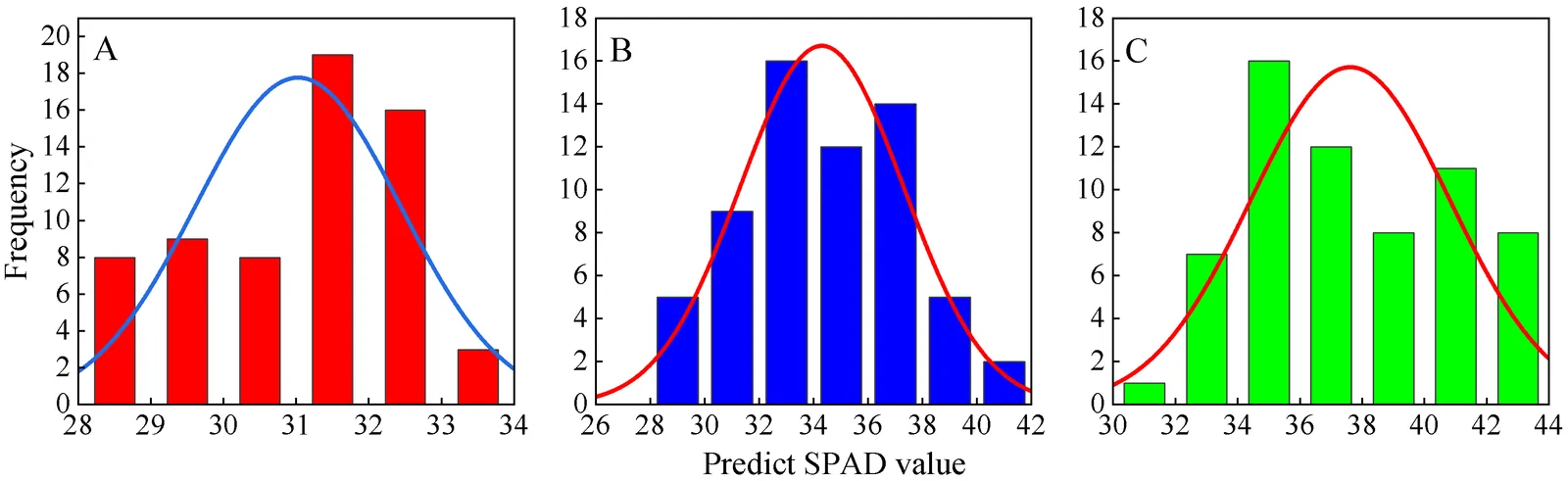
Histogram of predicted SPAD value distribution of maize at each growth stage. **(A)** V6 stage; **(B)** VT stage; **(C)** R3 stage.

**Figure 11** shows the frequency distribution of the predicted maize SPAD values at the different growth stages. At the V6 stage, the predicted SPAD values ranged from 28 to 34, with 31–33 being the most frequent; at this stage, the plants were approximately 1 m tall and in a period of rapid growth (**Figure 11A**). At the VT stage, the predicted values ranged from 26 to 42, with 32–36 being the most frequent. During this period, the plants were approximately 2.2 m tall and at the reproductive stage (**Figure 11B**). At the grain-filling stage, the predicted values ranged from 30 to 44, with 34–42 being the most frequent; the plants were approximately 2.6 m tall (**Figure 11C**).

## 4 Discussion

### 4.1 Effects of different treatments on maize SPAD values

The SPAD value of maize is an important physiological indicator that plays a key role in photosynthesis, nutrient uptake, and growth and development (Shivashankar et al., 2025). Chlorophyll is the main pigment driving photosynthesis; it absorbs light energy and converts it into chemical energy, thereby promoting plant growth (Pareek et al., 2017). In this study, a field experiment under subsurface drip irrigation was conducted to examine the effect of water-N coupling on maize SPAD values. As the growth season progressed, the SPAD value generally increased and then decreased, and with increasing nitrogen rate, the chlorophyll content first increased and then declined. This is consistent with findings for crops such as maize and wheat (Li et al., 2022; Shivashankar et al., 2025) and confirms the physiological mechanism whereby moderate nitrogen can promote chlorophyll synthesis, whereas excessive nitrogen disrupts nutrient metabolism and inhibits chlorophyll accumulation (Zhou et al., 2025). Notably, in the Yellow River irrigation area, where annual precipitation is only 168 mm, the combination of medium water (W2, 225 m³/hm²) and medium nitrogen (N2, 210 kg/hm²) was more favorable for stable chlorophyll accumulation in maize leaves, with the SPAD value reaching 38.32 at the grain-filling stage. This differs from the optimal water-N ratio reported for humid regions, reflecting the specific response of crops in arid irrigation areas to water-N coupling and providing key parameters for precise water and fertilizer management of maize in this region.

### 4.2 Multi-feature combination, model optimization and cross-device calibration improve SPAD estimation accuracy

A sound selection of feature variables is central to improving the model accuracy (Eskandari et al., 2020). At the feature level, the combined spectral (SI) and texture (TI) variables achieved notably higher accuracy, with an R^2^ at least 9% greater than that of either feature type alone. This advantage arises from the complementarity of the two feature types (Kang et al., 2024; Yuan et al., 2013): spectral features directly quantify leaf light absorption and reflection properties at different wavelengths. For example, the red-edge band RE2 and NDVI both had absolute correlations with SPAD greater than 0.4, making them core indicators of crop physiological status, whereas the texture features, by describing the spatial distribution of canopy pixels, indirectly reflect canopy uniformity and density, and thus supply structural information that spectral features capture poorly. The combination of these two characteristics provides a more comprehensive understanding of maize growth (Xie et al., 2023; Zhang et al., 2022). At the model level, the RF model showed the best predictive performance owing to its ensemble structure, which reduces overfitting through the voting of multiple decision trees while effectively handling nonlinear feature relationships and high-dimensional redundancy. This is consistent with the adaptive advantages of RF reported for estimating vegetation physiological parameters (Elavarasan and Vincent, 2021). In terms of technical innovation, a UAV–smartphone feature calibration matrix was constructed using regularized least squares, and the cross-device bias was corrected using a residual-compensation term that accounts for the inherent differences in spectral range and photosensitivity between devices and for the nonlinear coupling among features. After calibration, the smartphone SPAD estimation R² reached 0.80, 0.86, and 0.82 at the V6, VT, and R3 stages, respectively, effectively reconciling smartphone portability with the measurement accuracy. Compared with conventional direct feature mapping, this approach offers clear advantages and provides a new route for rapid field-based SPAD monitoring (Hu et al., 2024).

The proposed framework showed stable performance across different water-deficit treatments and maize growth stages. RF consistently outperformed RR and SVM, and smartphone-based SPAD estimation after cross-device calibration achieved R² values of 0.80–0.86 across the three growth stages. These results support the stability of the framework under standardized field conditions, although its robustness under more variable illumination and leaf-posture conditions requires further validation.

### 4.3 Research limitations and future research directions

Several limitations of this study point to directions for future research. First, the potential effects of UAV flight parameters such as flight altitude and heading overlap were not quantified. A single configuration was used throughout (20 m altitude and 80% heading and side overlap), without testing parameter gradients, such as altitudes of 10–30 m or overlaps of 60%-90%, and their effects on image spatial resolution, mosaicking accuracy, and spectral and texture feature extraction. Second, UAV–smartphone spectral calibration requires further study. Although a feature mapping matrix was built using regularized least squares with residual compensation, the calibration was based mainly on natural field lighting and did not consider extreme conditions, such as strong specular reflection, low light, or variable leaf posture, which interfere with the cross-device feature consistency. In addition, the applicability of this method to other regions has not been examined. Future research could set different flight altitudes to assess their effect on the accuracy of maize growth indicator estimation and build models linking growth stage, canopy cover, and flight parameters to identify the optimal parameter combination for each stage and further reduce systematic errors during data acquisition. In addition, deep learning algorithms can replace the conventional regularized least squares method to automatically learn the nonlinear mapping between UAV and smartphone features and reduce the limitations of a manually designed calibration matrix.

It should be noted that UAV images represent canopy-level mixed pixels (containing leaves, shadows, and gaps), whereas smartphone images represent pure leaf-level pixels. Rather than aligning individual pixels, the calibration framework pairs UAV features extracted from a 3×3 neighborhood around each GPS-tagged point with the averaged leaf-region values from the corresponding smartphone image of the same plant, ensuring that both feature vectors describe the same individual. Moreover, the regularized least-squares framework learns a statistical mapping at the feature level, inherently accommodating scale-induced discrepancies through the residual-compensation term. Nevertheless, structural information in canopy mixed pixels, such as shadows and gaps, is absent from leaf-level images, and future work may incorporate canopy cover or leaf area index as auxiliary variables to address this scale mismatch.

## 5 Conclusion

This study used multispectral imagery from an unmanned aerial vehicle (UAV) together with ground-measured SPAD data to extract vegetation indices and texture features, and combined several machine learning models to estimate the SPAD values of maize under different water and fertilizer levels in the Yellow River irrigation district. The effects of growth stage, water and fertilizer conditions, and soil background on the estimation accuracy were examined in detail. After cross-device (camera) calibration, the optimal model successfully estimated the SPAD values at each growth stage. The main conclusions are as follows:

1. Optimal irrigation and nitrogen management is critical for maintaining maize chlorophyll content under the arid conditions of the Yellow River irrigation district. Our results indicate that moderate irrigation (225 m³/ha) combined with medium nitrogen application (210 kg/ha) is most favorable for sustaining high SPAD levels during grain filling, providing a practical water-fertilizer strategy for local farmers.
2. The integration of spectral indices and texture features from UAV imagery substantially improves SPAD estimation accuracy. Among the models evaluated, Random Forest consistently outperformed RR and SVM across all growth stages, demonstrating its strong capability to capture nonlinear relationships between remote sensing features and crop physiological status.
3. The proposed cross-device calibration framework effectively bridges the spectral gap between smartphone RGB and UAV multispectral data, achieving reliable SPAD estimation from smartphone-derived features (R² = 0.80–0.86). This provides a low-cost and accessible alternative to UAV-based monitoring, enabling rapid crop nitrogen status assessment and supporting timely water-fertilizer management decisions in arid irrigation districts.

## Data Availability

The original contributions presented in the study are included in the article/supplementary material. Further inquiries can be directed to the corresponding author.
